# Carbapenem Resistance in *Acinetobacter baumannii*: Mechanisms, Therapeutics, and Innovations

**DOI:** 10.3390/microorganisms13071501

**Published:** 2025-06-27

**Authors:** Joyce de Souza, Helena Regina Salomé D’Espindula, Isabel de Farias Ribeiro, Geiziane Aparecida Gonçalves, Marcelo Pillonetto, Helisson Faoro

**Affiliations:** 1 Laboratory for Applied Science and Technology in Health, Carlos Chagas Institute, Oswaldo Cruz Foundation FIOCRUZ, Curitiba 81350-010, Brazil; 2 Molecular Bacteriology Section, Laboratório Central de Saúde Pública do Estado LACEN-PR, Curitiba 83060-500, Brazilmarcelopilonetto@gmail.com (M.P.)

**Keywords:** *Acinetobacter baumannii*, carbapenem resistance, beta-lactamases, alternative therapies, phage therapy, efflux pumps, antimicrobial peptides, artificial intelligence

## Abstract

The global rise of carbapenem-resistant *Acinetobacter baumannii* (CRAB) strains poses a critical challenge to healthcare systems due to limited therapeutic options and high mortality rates, especially in intensive care settings. This review explores the epidemiological landscape and molecular mechanisms driving carbapenem resistance, including the production of diverse beta-lactamases (particularly OXA-type enzymes), porin loss, efflux pump overexpression, and mutations in antibiotic targets. Emerging treatment strategies are discussed, such as the use of new beta-lactam–beta-lactamase inhibitor combinations (e.g., sulbactam–durlobactam), siderophore cephalosporins, next-generation polymyxins, as well as novel agents like zosurabalpin and rifabutin (BV100). Alternative approaches—including phage therapy, antimicrobial peptides, CRISPR-based gene editing, and nanoparticle-based delivery systems—are also evaluated for their potential to bypass traditional resistance mechanisms. Furthermore, advances in artificial intelligence and multi-omics integration are highlighted as tools for identifying novel drug targets and predicting resistance profiles. Together, these innovations represent a multifaceted strategy to overcome CRAB infections, yet their successful implementation requires further clinical validation and coordinated surveillance efforts. This analysis highlights the urgent need for continued investment in innovative treatments and effective resistance monitoring to limit the spread of CRAB and protect the effectiveness of last-line antibiotics.

## 1. Emergence of Carbapenem-Resistant *A. baumannii* (CRAB)

**Figure 1:**
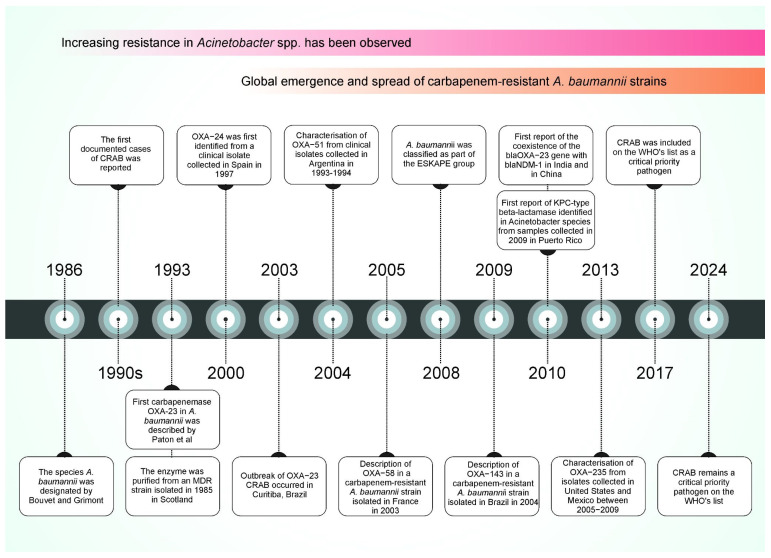
The timeline of key publications related to carbapenem resistance in *A. baumannii*. The figure highlights the first reports of major carbapenemases being identified in *A. baumannii* and the evolution of the species into a globally recognised critical pathogen. Abbreviations: CRAB, carbapenem-resistant *Acinetobacter baumannii*; MDR, multidrug-resistant; ESKAPE, *Enterococcus faecium*, *Staphylococcus aureus*, *Klebsiella pneumoniae*, *Acinetobacter baumannii*, *Pseudomonas aeruginosa*, *Enterobacter* spp., and *Escherichia coli*; WHO, World Health Organisation. References corresponding to events in the timeline: 1986 [4]; 1990s [3]; 1993 [5]; 2000 [6]; 2003 [7]; 2004 [8]; 2005 [9]; 2008 [10]; 2009 [11]; 2010 [12]; 2013 [13]; 2017 [14]; 2024 [15].

During the early 1970s, *Acinetobacter* spp. isolates were generally susceptible to multiple antibiotic classes, including gentamicin, minocycline, nalidixic acid, ampicillin, and carbenicillin, either as monotherapy or in combination. However, since 1975, increasing resistance has been observed across nearly all antimicrobial groups, progressively narrowing the therapeutic options. By the late 1980s and 1990s, the global emergence and dissemination of *Acinetobacter* strains resistant to imipenem had significantly restricted the treatment alternatives. Carbapenems remained the only effective agents against multidrug-resistant (MDR) *A. baumannii* infections. The first documented case of CRAB was reported in 1991 [1,2], and in the following years, resistance rates continued to rise, posing a growing challenge to clinical management [3]. The general timeline of resistance emergence is illustrated in Figure 1.

A systematic review analysing CRAB infections in intensive care units (ICUs) across the WHO-defined regions of Europe (EUR), the Eastern Mediterranean (EMR), and Africa (AFR) reported a pooled incidence of 41.7 cases per 1000 patients (95% CI 21.6–78.7) and an incidence density of 2.1 cases per 1000 patient-days (95% CI 1.2–3.7). Additionally, CRAB strains were responsible for 13.6% (95% CI 9.7–18.7) of all hospital-acquired infections in ICUs, underscoring their clinical significance in these regions [16]. However, the global epidemiology of *A. baumannii* infections (including those caused or not by carbapenem-resistant strains) is highly heterogeneous. The latest ECDC point prevalence survey (2022–2023) highlights this variation across European countries, with *A. baumannii* comprising as much as 17.6% of hospital-associated infections in Kosovo but less than 0.1% in Belgium, Ireland, Luxembourg, Malta, and the Netherlands [17]. Such discrepancies are influenced by multiple factors, including the climate, infection control infrastructure, and antibiotic stewardship practices. *A. baumannii* is notably clonal and prone to outbreak-driven dissemination, often flourishing in settings with limited resources or suboptimal infection prevention strategies. This regional variability is further reflected in a systematic review of resistance patterns in the Middle East and South Asia (2012–2022), where high rates of carbapenem resistance were observed in bloodstream infections, with a prevalence of 0.95 (95% CI: 0.92–0.97, *n* = 1193) in Türkiye, 0.82 (95% CI: 0.75–0.88, *n* = 159) in Iran, and 0.65 (95% CI: 0.17–1.00, *n* = 240) in Pakistan [18]. Also, according to the latest available data from the WHO Global Antimicrobial Resistance and Use Surveillance System (GLASS) dashboard (2022), *Acinetobacter* spp. bloodstream infections (BCIs) exhibited a high rate of carbapenem resistance, with 40,657 out of 45,485 cases (89%) showing resistance [19].

Whole-genome-based approaches to molecular epidemiology and the global distribution of *A. baumannii* show that the international clone (IC) 2 constituted the largest and most widely spread *A. baumannii* clonal lineage. It is most prevalent in Africa, Asia, Europe, Oceania, and North America [20,21]. An analysis of more than 15,000 publicly available genomes has recently suggested that an epidemic super-lineage has arisen among isolates assigned to international clone 2 (IC2) [21]. Notably, unlike in other regions, IC5 and IC7 are the most prevalent in South America [20,21]. Acquired carbapenemase genes were identified in approximately 66.9% to 92.2% of *A. baumannii* isolates globally, with *bla*_OXA-23-like_ and *bla*_OXA-40-like_ genes being the most prevalent [22,23]. Alarmingly, the global prevalence of carbapenemase genes seems to have risen over time, increasing from 66% of the isolates collected between 2000 and 2014 to 96.2% of the isolates collected in 2024. This rise may partially result from a bias towards sequencing carbapenemase-positive isolates [22].

## 2. Molecular Mechanisms of Carbapenem Resistance in *A. baumannii*

**Figure 2:**
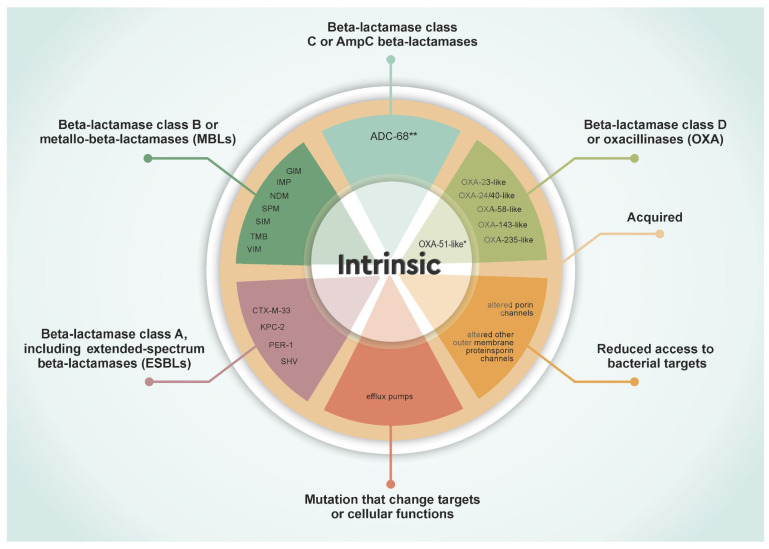
Schematic representation of the main mechanisms underlying carbapenem resistance in *A. baumannii*. * The *bla_OXA-51_* gene is intrinsic to *A. baumannii* and has limited carbapenemase activity, but its expression increases significantly when associated with upstream IS elements. ** Among the class C β-lactamases identified in *A. baumannii*, ADC-68 is, to date, the only variant experimentally confirmed to possess carbapenemase activity.

CRABs may arise from a combination of enzymatic degradation, reduced membrane permeability, active antibiotic efflux, and outer membrane modifications. These mechanisms, often acting synergistically, contribute to the pathogen’s ability to survive even in the presence of last-line antimicrobials [24]. Understanding the molecular basis of resistance is central for developing targeted therapeutic strategies [25]. An overview of these resistance mechanisms in CRAB is illustrated in Figure 2.

### 2.1. Beta-Lactamases

Beta-lactamases hydrolyse beta-lactam antibiotics, including carbapenems, conferring resistance. *A. baumannii* harbours multiple classes of beta-lactamases. Here we used Ambler (molecular) classification, including class A (including the extended-spectrum beta-lactamases—ESBLs), class B (or metallo-beta-lactamases—MBLs), class C (or AmpC beta-lactamases), and class D (or OXA-type) [26]. The primary carbapenem resistance genes identified in *A. baumannii* are listed in Table 1 and Table 2.

#### 2.1.1. Class A Beta-Lactamases, Including Extended-Spectrum Beta-Lactamases (ESBLs)

Although less common in *A. baumannii*, class A beta-lactamases such as PER, CTX-M, and KPC have been sporadically reported, further complicating treatment options. PER-1 was one of the first ESBLs identified in *A. baumannii*, particularly in isolates from Turkey and Europe, and has been associated with increased resistance in multidrug-resistant strains [27,28]. Notably, PER-1 has also been linked to reduced susceptibility to cefiderocol, likely due to its high ceftazidimase activity, given the structural similarities between ceftazidime and cefiderocol [29,30]. The first detection of KPC (*Klebsiella pneumoniae* carbapenemase) in *A. baumannii* was reported in Colombia, highlighting the potential for interspecies gene transfer between *Klebsiella pneumoniae* and *A. baumannii* through mobile genetic elements, such as plasmids and transposons [31,32]. Variants of the CTX-M family have also been sporadically detected in *A. baumannii*, indicating possible horizontal gene transfer from *Enterobacterales* [24].

In addition, SHV-family beta-lactamases, although primarily found in *Klebsiella pneumoniae*, have been reported in carbapenem-resistant *A. baumannii* isolates in Iran and China [33,34]. Similarly, TEM-family beta-lactamases, which are widely distributed among Enterobacterales, have also been detected in *A. baumannii* isolates in China [34]. However, further studies are needed to determine the exact impact of SHV and TEM enzymes on carbapenem resistance in this species, as their presence may be linked to co-selection with other resistance determinants, rather than the direct hydrolysis of carbapenems.

**Table 1:** Gene families and resistance mechanisms associated with molecular class A (including extended-spectrum beta-lactamases, ESBLs), class B (metallo-beta-lactamases, MBLs), and class C carbapenemases, with an emphasis on those previously reported in *Acinetobacter baumannii*.

Ambler Molecular and Structural Classification	Gene Family	Accession *	e.g., Genes	References
A	CTX-M beta-lactamase	ARO:3000016	CTX-M-33	[35,36]
A	KPC beta-lactamase	ARO:3000059	KPC-2	[31,32]
A	PER beta-lactamase	ARO:3000056	PER-1	[27,28]
A	SHV beta-lactamase ***	ARO:3000015	SHV-12	[33,34,37]
A	TEM beta-lactamase **	ARO:3000014	TEM-1	[34]
A	VEB beta-lactamase	ARO:3000043	VEB-1	[38,39]
B or MBLs	GIM beta-lactamase	ARO:3003195	GIM	[40]
B or MBLs	IMP beta-lactamase	ARO:3000020	IMP-1, IMP-11, IMP-2, IMP-4, IMP-5, IMP-6, IMP-69	[41,42,43,44,45,46,47,48]
B or MBLs	NDM beta-lactamase	ARO:3000057	NDM-1, NDM-2	[49]
B or MBLs	SIM beta-lactamase	ARO:3004206	SIM-1	[41]
B or MBLs	SPM beta-lactamase	ARO:3000580	SPM	[50]
B or MBLs	TMB beta-lactamase	ARO:3004104	TMB-2	[51]
B or MBLs	VIM beta-lactamase	ARO:3000021	VIM-2	[52]
C or para Cephalosporins	ADC beta-lactamase with carbapenemase activity	ARO:3004545	ADC-68 ***	[53]

* Accession number in CARD—The Comprehensive Antibiotic Resistance Database. ** Further studies are needed to determine the exact impact of SHV and TEM enzymes on carbapenem resistance. *** Among the class C β-lactamases identified in *A. baumannii*, ADC-68 is, to date, the only variant experimentally confirmed to possess carbapenemase activity.

#### 2.1.2. Class B Beta-Lactamases or MBLs

MBLs, such as NDM-1, require zinc ions for their catalytic activity and confer resistance to nearly all beta-lactams, except for monobactams. Additionally, the NDM family’s role in resistance to the recently FDA-approved cefiderocol appears to reduce susceptibility with an increase in MIC values [29,54]. The first reports of NDM-producing *A. baumannii* emerged in 2010 in India [12] and in 2011 in China [55]. The *bla*_NDM_ genes, carried on mobile genetic elements, have facilitated rapid global dissemination [56]. Environmental Acinetobacter species act as reservoirs for *bla*_NDM_, promoting its spread [57,58,59].

#### 2.1.3. Class C Beta-Lactamases

Class C beta-lactamases generally do not hydrolyse carbapenems effectively and, therefore, have a limited role in CRAB. To the best of our knowledge, ADC-68 remains the only class C beta-lactamase identified in *A. baumannii* to date that has been experimentally confirmed to exhibit carbapenemase activity [53].

**Table 2:** Gene families and resistance mechanisms associated with molecular class D beta-lactamases (also known as CHDLs or oxacillinases), with an emphasis on those previously reported in *Acinetobacter baumannii*.

Gene Family	Variantes *	Accession **	Associated Insertion Sequence Elements	Plasmid or Chromosomal	References
OXA-23-like beta-lactamase	52	ARO:3007710	ISAba1 and ISAba4	Chromosomal or Plasmid	[60]
OXA-24-like beta-lactamase (or OXA-40-like beta-lactamase)	14	ARO:3007711	ISAba1	Chromosomal or Plasmid	[61]
OXA-48-like beta-lactamase	67	ARO:3007721	ND	ND	[62,63,64]
OXA-51-like beta-lactamase	383	ARO:3007725	ISAba1, ISAba2, ISAba825, ISAba15, ISAba16, ISAba19	Chromosomal	[60,65]
OXA-58-like beta-lactamase	8	ARO:3007728	ISAba1, ISAba2, ISAba3, ISAba125 and IS18	Chromosomal or Plasmid	[65,66]
OXA-134-like beta-lactamase	28	ARO:3007700	ND	ND	[62,65]
OXA-143-like beta-lactamase	11	ARO:3007701	ND	Plasmid	[11,62]

ND—not determined. * Sub-term(s) ontology in CARD—The Comprehensive Antibiotic Resistance Database. ** Accession number in CARD—The Comprehensive Antibiotic Resistance Database.

#### 2.1.4. Class D Beta-Lactamases or OXA-Type

OXA-type carbapenemases are the most prevalent in *A. baumannii*. The first imipenem-resistant strain was reported in Scotland in 1985 [5] and was later identified as OXA-23 [67]. OXA-23 is globally disseminated and associated with major outbreaks, such as that in Brazil in 2003 [7]. Since then, multiple variants of these enzymes have been identified globally, with reports from Scotland, Spain, France, Japan, Singapore, China, Brazil, Cuba, and Kuwait [8]. Other OXA-type carbapenemases, such as OXA-24/40-like, OXA-58, and OXA-143-like, have also emerged, contributing to regional resistance patterns [3].

It is important to highlight that the dissemination and expression of OXA-type carbapenemases in *A. baumannii* are strongly influenced by associated insertion sequence (IS) elements. These mobile genetic elements, such as *ISAba1* and *ISAba3*, can enhance OXA gene expression by providing strong promoter sequences, leading to increased carbapenem resistance. A notable example is *bla*_OXA-51_, which is intrinsically present in *A. baumannii* with limited carbapenemase activity. However, when associated with *ISAba1* upstream, its expression is significantly upregulated, contributing to high-level carbapenem resistance. Additionally, IS elements facilitate horizontal gene transfer, promoting the spread of *bla*_OXA_ variants across different strains and geographic regions, further complicating infection control and treatment strategies [65].

Also, a bioinformatics analysis of sequenced beta-lactamases revealed that approximately 60% of class D OXA enzymes contain a lipobox sequence in their signal peptide, which promotes lipidation and anchoring to the bacterial outer membrane. Experimental evidence confirmed that OXA-23 and OXA-24/40 are membrane-bound proteins in *A. baumannii*, contrasting with soluble beta-lactamases, such as OXA-48, commonly found in Enterobacterales. Furthermore, outer membrane vesicles (OMVs) released by *A. baumannii* were shown to contain active OXA-23 and OXA-24/40, conferring protection not only to *A. baumannii* itself but also to *Escherichia coli* and *Pseudomonas aeruginosa* against piperacillin and imipenem. These findings suggest that lipidation and OMV-mediated transport may play a crucial role in antimicrobial resistance dissemination, particularly in polymicrobial infections where *Acinetobacter* spp. are frequently involved. Understanding the cellular localisation of beta-lactamases is, therefore, essential for developing effective therapeutic strategies to counteract resistance [68].

#### 2.1.5. Other Gene Families

Besides these gene families, it is important to acknowledge the potential existence of additional carbapenemase-encoding genes that have not yet been reported in *A. baumannii*. Their absence in current isolates may reflect either a genuine lack of these genes or insufficient targeted research. Several carbapenemase genes previously identified in other Gram-negative bacteria, particularly in Enterobacterales, pose a theoretical risk of gene transfer to *A. baumannii*. Examples include *bla*_BKC_ (ARO:3004756), *bla*_GES_ (ARO:3000066), *bla*_IMI_ (ARO:3000018), *bla*_SME_ (ARO:3000055), *bla*_OXA-114-like_ (ARO:3007698), *bla*_OXA-198-like_ (ARO:3007703), and numerous others. Continued genomic surveillance is essential to detect any future emergence of these or other novel carbapenemase genes in *A. baumannii*.

### 2.2. Target Modifications and Reduced Antibiotic Access

Beyond beta-lactamase production, *A. baumannii* can evade antibiotic action through various mechanisms that may act independently or in combination. These include limiting drug entry via porin loss and outer membrane (OM) modifications, as well as actively expelling antibiotics through efflux pumps. The interplay of these strategies contributes to reduced intracellular antimicrobial concentrations and the development of multidrug resistance.

#### 2.2.1. Low Permeability of Its Outer Membrane (OM)

Gram-negative bacteria possess a bilayer membrane structure, which serves as a selective barrier to antibiotic entry. Porins mediate passive antibiotic diffusion into bacterial cells, but *A. baumannii* has inherently low outer membrane permeability due to the absence of large general porins, such as OmpF and OmpC. The loss or modification of outer membrane proteins (OMPs) can further restrict antibiotic entry [69].

The CarO (carbapenem-associated outer membrane protein) has been associated with carbapenem uptake [70], but recent crystal structure analyses of three isoforms of CarO indicate that it primarily facilitates the transport of small amino acids, rather than beta-lactams [69].

Due to the absence of large-channel porins, *A. baumannii* relies on alternative outer membrane proteins for small-molecule transport. A recent study proposed that outer membrane carboxylate channels (Occs, formerly known as OprDs) may play a key role in this process, including the uptake of antibiotics. The genome of *A. baumannii* strains, such as ATCC 17978 and AB307-0294, encodes five Occ orthologs. In a recent publication, four of these channels (OccAB1 to OccAB4) were successfully expressed, purified, and structurally characterised by X-ray crystallography. The resulting structures revealed open pores of varying diameters, with OccAB1 exhibiting the widest channel. Functional analyses using electrophysiology and liposome swelling assays showed that OccAB1 has the highest conductance and the most efficient uptake of small molecules. Importantly, OccAB1 also demonstrated the greatest in vitro permeability for the carbapenem antibiotics imipenem and meropenem, supporting its potential role in carbapenem influx [69].

#### 2.2.2. Altered Molecular Antibiotic Targets

Penicillin-binding proteins (PBPs) are key enzymes in bacterial cell wall synthesis and the primary targets of beta-lactam antibiotics, including carbapenems. While PBP modifications have been implicated in carbapenem resistance in *A. baumannii*, their role remains poorly understood.

A study analysing *A. baumannii* clinical isolates in Spain identified the allelic variations of seven PBP genes and one monofunctional transglycosylase (MGT) gene. While most allelic variations resulted in silent mutations, an insertion sequence disrupting PBP6b was found in an endemic carbapenem-resistant clone. This suggests that genetic rearrangements in PBP-encoding genes may contribute to resistance, though no direct correlation was established between specific PBP mutations and beta-lactam susceptibility [71].

Another study analysing *A. baumannii* bloodstream isolates found that carbapenem-resistant strains produced additional beta-lactamases (pI 6.3 and 7.0), which contributed to the increased hydrolysis of imipenem and meropenem. These isolates also exhibited the loss of a 22.5 kDa outer membrane protein (OMP), reducing antibiotic permeability, and the absence of the 73.2 kDa PBP2 band, impairing carbapenem binding. These findings highlight the interplay between beta-lactamase production, porin loss, and PBP modifications in carbapenem resistance [72].

A separate study identified PBP 7/8 as a critical factor for bacterial survival and virulence in *A. baumannii*. Its loss increased membrane permeability, making the bacteria more susceptible to complement attack, lysozyme activity, and various antimicrobials. Additionally, PBP 7/8 deletion led to reduced lipid A, increased cell aggregation, and a shift to a coccoidal morphology. In a mouse pneumonia model, its absence reduced bacterial lethality 11-fold, highlighting PBP 7/8 as a promising drug target for CRAB [73].

A systematic review found that 31.7% of *A. baumannii* isolates resistant to the sulbactam–durlobactam combination, a new antibiotic combination for CRAB, have substitutions in PBP3, typically near their active serine site (S336) [74]. This further emphasises the role of specific PBP modifications in undermining the efficacy of beta-lactam-based therapies, even among novel drug combinations.

#### 2.2.3. Efflux Pumps

Efflux pumps in *A. baumannii* are typically organised as tripartite systems composed of an outer membrane channel, a periplasmic adaptor protein, and an inner membrane transporter. These complexes facilitate the extrusion of antibiotics and toxic compounds from the cell. These transport systems can work synergistically with other resistance mechanisms, such as porin loss and beta-lactamase production, further compromising treatment options. Four main families of efflux pumps have been linked to antimicrobial resistance in *A. baumannii*: the Major Facilitator Superfamily (MFS), the Resistance-Nodulation-Division (RND) family, the Small Multidrug Resistance (SMR) family, and the Multidrug and Toxic Compound Extrusion (MATE) family [75].

Among them, the RND systems have the broadest substrate ranges. Specifically, AdeABC, AdeFGH, and AdeIJK have been associated with multidrug resistance in *A. baumannii*. A study investigating MDR *A. baumannii* clinical isolates demonstrated resistance to carbapenems by the production of beta-lactamases and the overexpression of RND-type efflux pumps, particularly AdeABC. Among 14 genetically distinct MDR isolates, the adeB gene (AdeABC) was detected in 13, and its overexpression was observed in 10 strains. In contrast, only the moderate expression of AdeFGH was detected in seven isolates, and none showed the overexpression of AdeIJK. Mutational analyses revealed functional mutations in the AdeRS two-component regulatory system in all the strains overexpressing AdeABC, with two mutational hotspots suggesting convergent evolution and possible horizontal gene transfer. These findings corroborate the proposed contribution of AdeABC and its regulatory mutations to efflux-mediated resistance in clinical *A. baumannii* isolates, although further studies are needed to fully elucidate their specific role in resistance to carbapenems [76].

Another study evaluating the impact of imipenem exposure on *A. baumannii* revealed that this carbapenem may act as a strong inducer of multidrug resistance. Imipenem-selected mutants developed from susceptible strains showed increased resistance profiles, associated with the altered expression of OXA-51-like carbapenemase, efflux pumps, and AmpC beta-lactamase. Notably, the use of efflux pump inhibitors (CCCP and NMP) partially restored the susceptibility to imipenem and amikacin, suggesting the involvement of active efflux in the resistance phenotype [77].

## 3. Promising Therapeutic Innovations Against CRAB

### 3.1. New Antibiotics

Cefiderocol is an alternative treatment for CRAB and was approved by the Food and Drug Administration (FDA) in 2019 [78] and the European Medicines Agency (EMA) in 2020 [79]. Treatment with cefiderocol is indicated by the Infectious Diseases Society of America (IDSA) and the European Society of Clinical Microbiology and Infectious Diseases (ESCMID) in combined antibiotic therapies to enhance its action through synergy with other antibiotics [80,81]. This antibiotic belongs to the cephalosporin class and has siderophore activity, which means it utilises the mechanisms that bacteria use to absorb iron to penetrate their cell membranes. Once the drug enters the bacterial cell, it binds to PBPs, inhibiting the production of cell wall peptidoglycans [82]. Meta-analyses including data from randomised controlled trials (RCTs) and observational studies have demonstrated that cefiderocol-based treatment, compared to the best available therapy, which was generally colistin-based, was associated with a significantly lower risk of death in patients with CRAB infections [83,84]. Additionally, higher cure rates and lower nephrotoxicity were observed with cefiderocol compared to colistin-based treatments [85].

In addition to cefiderocol, whose clinical use is relatively recent, several other molecules with therapeutic potential for treating CRAB infections have been identified and are currently under investigation. However, unlike cefiderocol, most of these new agents remain in the early stages of development and have not yet received clinical approval. Further studies and clinical trials are necessary to establish their efficacy and safety. Below, we highlight some of these promising candidates.

Among the promising new therapies is a clinical candidate derived from tethered macrocyclic peptides (MCPs), known as zosurabalpine (RG6006). This compound has demonstrated efficacy against CRAB isolates both in vitro and in murine models of infection [86]. The mechanism of action of these molecules involves inhibiting the LptB_2_FGC complex, which is responsible for transporting lipopolysaccharide (LPS) molecules through the periplasmic space to the outer membrane. By binding to this complex, zosurabalpine causes an accumulation of LPS within the bacterial cell, which is lethal to the bacteria [86,87]. A phase I clinical trial demonstrated the agent’s safety and tolerability, along with a favourable pharmacokinetic profile in healthy participants [87,88].

Another new antibiotic is BV100, a parenteral formulation of rifabutin, a spiropiperidyl rifamycin analog. Rifabutin is unique compared to rifamycins, which often show reduced effectiveness against Gram-negative organisms. Unlike these other compounds, rifabutin is actively transported by the FhuE siderophore receptor across the outer membrane of *A. baumannii*. This transport leads to high intracellular concentrations of rifabutin, inhibiting RNA polymerase [89]. The intravenous formulation BV100 aims to reduce the low bioavailability often associated with oral formulations, which can lead to subtherapeutic drug concentrations. BV100 has demonstrated remarkable in vivo activity against CRAB [89,90]. A phase I trial of BV100 has confirmed the drug’s safety and tolerability. In phase II of the study (Clinicaltrials.gov ID NCT05685615), patients were randomly assigned to receive either BV100 in combination with polymyxin or the best available therapy (BAT). The BV100 treatment was safe and well tolerated, with no drug-related serious adverse events. Patients in the BV100 combination had lower 14-day and 28-day mortality rates (12.5% and 25%) compared to the BAT arm (40% and 60%). Microbiological response and clinical cure rates were higher in the BV100 arm (75%) than in the BAT arm (50% and 30%, respectively) [91]. The findings from this trial will provide insight into the potential benefits of combining rifabutin with polymyxin to prevent the emergence of resistance [87].

Molecules derived from or modified from other classes of traditional antibiotics have been explored in order to find effective alternatives against resistant bacteria and expand the available therapeutic arsenal. One such compound is TP-6076, a synthetic tetracycline belonging to the fluorocycline class. Changes in the molecular structure of the compound concerning current tetracyclines showed better or comparable activity to other tetracyclines, ampicillin–sulbactam, and colistin, among others, against CRAB [92,93,94]. The molecules identified as SPR206, QPX9003, FADDI-002, and FADDI-003 are next-generation or derivative polymyxins that have demonstrated potential in treating CRAB in both laboratory and animal studies. These new compounds typically display a better activity and safety profile compared to traditional polymyxins [95]. Among antibiotic adjuvants, SPR741 stands out as a cationic peptide derived from polymyxin B, which, after undergoing a process of change in the hydrophobic fatty acyl tail, had its nephrotoxicity reduced [92]. The drug acts on the cell membrane of some bacteria to increase permeability, thus enhancing the action of other antibiotics [93]. The adjuvant was found to be ineffective against Gram-negative bacteria when used as monotherapy [92].

Additionally, lariocidin, a lasso peptide antibiotic produced by *Paenibacillus* sp. M2, has recently been identified and demonstrated effective activity in a mouse model of CRAB infection [96]. However, this new molecule has not yet received clinical approval and still requires additional clinical trials.

### 3.2. Combination Therapies

The treatment of infections caused by *A. baumannii* can be particularly challenging due to its remarkable ability to acquire multiple antibiotic resistance mechanisms. Using antibiotic combinations may help reduce the risk of developing additional resistance during treatment. Some of these combinations have demonstrated synergy in vitro, increasing efficacy against resistant strains. However, available clinical evidence regarding the superiority of combination therapies is still limited. Nevertheless, they continue to be a valid option, particularly in severe cases or when there are limited therapeutic options available [97,98]. A recommended approach for CRAB infections involves an antibiotic regimen that includes a sulbactam-containing agent. Both IDSA and ESCMID recommend a high-dose ampicillin–sulbactam regimen combined with at least one other agent, such as polymyxin B or tigecycline [80,81]. However, there is no consensus on its effectiveness [99,100,101,102]. In this context, other combination therapies may offer viable alternatives for treating CRAB.

#### 3.2.1. Sulbactam–Durlobactam

An alternative sulbactam combination therapy is sulbactam–durlobactam, a fixed-dose formulation recently approved only in the United States under the name Xacduro, for the treatment of hospital-acquired bacterial pneumonia (HABP) and ventilator-associated bacterial pneumonia (VABP) caused by isolates of the *Acinetobacter baumannii*–*calcoaceticus* complex [103,104,105]. In this combination, sulbactam acts as the active antibacterial agent, while durlobactam, a DBO-class serine-beta-lactamase inhibitor, protects sulbactam from hydrolysis by inhibiting class A, C, and D beta-lactamases [106,107]. Although durlobactam has no intrinsic bactericidal activity, its role in protecting sulbactam enhances the overall efficacy of the combination. An in vitro analysis of the sulbactam–durlobactam combination demonstrated activity comparable to colistin and superior to amikacin, minocycline, and sulbactam against CRAB isolates [108]. A clinical study indicates that the combination is effective against *A. baumannii* strains producing class D beta-lactamases, including OXA-type carbapenemases, which are responsible for high levels of carbapenem resistance [109]. The sulbactam–durlobactam combination demonstrated a superior patient recovery rate compared to colistin-based therapies, reaching approximately 62%, while colistin reached 40%. In addition, treatment with sulbactam–durlobactam was associated with lower mortality in CRAB infections and reduced nephrotoxicity [109]. However, the specific contribution of sulbactam–durlobactam alone, independent of co-administered carbapenems, has not been fully established [81,107]. Based on this and the available in vitro data, the current recommendation by the IDSA is that sulbactam–durlobactam be administered alongside imipenem–cilastatin or meropenem in the treatment of CRAB [81].

#### 3.2.2. Ceftolozane–Tazobactam

In addition to sulbactam and durlobactam, tazobactam is another beta-lactamase inhibitor that has been explored as a potential option for treating CRAB infections. The combination of ceftolozane and tazobactam, a novel cephalosporin–beta-lactamase inhibitor pair, has demonstrated significant synergistic effects when used alongside colistin. This combination may represent a promising alternative to colistin monotherapy in the management of CRAB infections [95].

#### 3.2.3. Synergistic Use of Polymyxinswith Tetracyclines or Glycopeptides

Tetracyclines (minocycline, tigecycline, and eravacycline) may demonstrate synergistic activity when combined with other classes like carbapenems or polymyxins in the treatment of CRAB. The combination of high-dose minocycline, polymyxin B, and sulbactam proved to be the most effective regimen in in vitro analyses against CRAB, when compared to monotherapy or double therapy using the same agents [110]. In murine models, the combination of minocycline with polymyxin B, rifampicin, or amikacin resulted in better outcomes for pneumonia compared to monotherapies [111,112,113]. Similar to minocycline, high-dose polymyxin B in combination with tigecycline, a minocycline derivative [114], resulted in greater reductions in the *A. baumannii* bacterial population compared with polymyxin alone in in vitro analysis [115]. Tigecycline, when combined with high-dose sulbactam, has been reported to effectively treat drug-resistant *A. baumannii* [99]. However, there is currently no consensus on the effectiveness of the treatment, whether as monotherapy or combination therapy with tigecycline, for clinical responses to CRAB infections [116]. Due to the lack of established susceptibility breakpoints for tigecycline for *A. baumannii*, the IDSA recommends the use of minocycline preferentially over tigecycline in combination treatments with other classes of antibiotics [81]. Eravacycline is a fluorocycline whose use for the treatment of serious intra-abdominal infections was approved in 2018 by the FDA [117]. Like other tetracyclines, eravacycline enters bacterial cells through porins, specifically the OmpA porin in *A. baumannii*. Once inside the bacteria, it binds to the 30S subunit of the ribosome, preventing the production of messenger RNA [118]. Additionally, the combination of cefiderocol and eravacycline shows promise as a therapeutic strategy for treating CRAB infections [119].

The combination of colistin with glycopeptides has shown a synergistic effect, likely due to the permeabilizing action of colistin on the bacterial outer membrane, which facilitates the entry of glycopeptide molecules that are normally excluded due to their large size [97]. In vitro studies demonstrated that the combination of colistin–vancomycin and the combination of colistin–doripenem–vancomycin both resulted in the complete killing of colistin-resistant *A. baumannii*. Similarly, in the *Galleria mellonella* infection model, these combinations significantly improved larval survival compared to monotherapies or other combinations [120]. Comparable results were observed for the combination of colistin–teicoplanin [121,122] and for the combination of colistin with the novel lipoglycopeptide telavancin [123,124]. Furthermore, a case report involving four patients with systemic infections caused by MDR *A. baumannii* showed that treatment with the colistin–vancomycin combination led to positive outcomes, with no infection relapses and no significant adverse events related to the concomitant administration [125].

#### 3.2.4. Efficacy and Challenges of Combination Therapies in Resistant Strains

Combination therapies for the treatment of CRAB aim to overcome resistance mechanisms and increase efficacy, but their challenges include inconsistency in outcomes and potential toxicity. Most of the evidence relies on retrospective observational studies, which adds a high risk of bias to them. Analyses of combination regimens generally come from low-certainty observational studies that analyse different antibiotic regimens, making it difficult to evaluate and indicate potential beneficial combinations [80]. In addition, there are still no susceptibility breakpoints determined by the Clinical and Laboratory Standards Institute (CLSI) or the European Committee on Antimicrobial Susceptibility Testing (EUCAST) for some cases, such as tigecycline and eravacycline. In others, it is necessary to re-evaluate the adjustment of minimum inhibitory concentration (MIC) breakpoints for high doses to optimise their use; for example, for ampicillin–subactam [126].

### 3.3. Development of New Carbapenemase Inhibitors

Considering that carbapenemases are enzymes capable of hydrolysing carbapenem antibiotics, they significantly compromise the efficacy of treatment against MDR infections. In this context, the development of carbapenemase inhibitors has emerged as a promising strategy to restore carbapenem activity.

Carbapenemase inhibitors based on cyclic (xeruborbactam/QPX7728) and bicyclic (taniborbactam/VNRX-5133) boronic acids have gained prominence as broad-spectrum inhibitors of serine- and metallo-beta-lactamases [127,128,129]. Although the main focus of many studies has been Enterobacterales [130], recent advances indicate that taniborbactam also has a potential effect against *A. baumannii* strains producing OXA-23 and OXA-24/40 [90]. Taniborbactam may be combined with cefepime [119,127] and xeruborbactam may be combined with cefiderocol [128] to enhance therapeutic synergy.

Diazabicyclooctane-based inhibitors (DBOs), such as relebactam and durlobactam, have been developed as inhibitors of class A and C beta-lactamases, but more recent studies explore their efficacy against class D carbapenemases in *A. baumannii* [131]. Durlobactam–sulbactam and imipenem–funobactam, in particular, have shown efficacy in combination with sulbactam and imipenem in combating the carbapenemases OXA-23, OXA-24/40, and OXA-58, which are predominant in *A. baumannii*. These combinations are already in advanced stages of clinical development and are considered a potential alternative for carbapenem-resistant infections [109,132]. Sulbactam–durlobactam has completed its clinical development program and has received regulatory approval in the United States [106]. Imipenem–cilastatin–funobactam has concluded its phase III clinical trial program but, to date, has only been submitted for regulatory review in China [133] and has not yet initiated patient recruitment for its planned global phase III trial targeting complicated urinary tract infections (Clinicaltrials.gov ID NCT05204368).

Another promising candidate in clinical trials is WCK-4234, a new beta-lactamase inhibitor of the DBO class. The drug has significant inhibitory activity against class A and D carbapenemases and class C enzymes, enhancing the activity of carbapenems against *A. baumannii* [134,135]. LN-1-255 is a beta-lactamase inhibitor drug derived from penicillanic acid sulfone that is capable of blocking carbapenem-hydrolysing class D beta-lactamases and is also active against class A and C beta-lactamases. The drug uses bacterial iron uptake pathways to enter the cell. In vitro tests have been evaluating whether the drug can help restore the efficacy of beta-lactam drugs, such as imipenem and meropenem [136,137].

### 3.4. Alternative and Innovative Therapies

The increasing prevalence of CRAB has driven the search for alternative therapeutic approaches beyond conventional antibiotics. Innovative strategies, including bacteriophage therapy, antimicrobial peptides, CRISPR-based interventions, plasmid-targeting approaches, and nanotechnology, offer promising avenues for overcoming antimicrobial resistance. These approaches aim to exploit novel mechanisms of action to circumvent existing resistance determinants and restore treatment efficacy.

#### 3.4.1. Development of New Efflux Pump Inhibitors

Given the critical role of efflux pumps in multidrug resistance, as previously discussed, considerable efforts have been directed toward the development of efflux pump inhibitors [138,139]. Unlike beta-lactamase inhibitors, there are currently no clinically developed or approved efflux pump inhibitors. However, some approaches, such as searching for compounds of natural origin and chemically modifying molecules for inhibitory effects, are in the prospecting stages. Natural plant compounds, such as alkaloids and flavonoids, and their chemically modified versions, have been extensively tested as potential efflux pump inhibitors [140]. Verma et al. [141] reported that naringenin dihydrochalcone-coated silver nanoparticles (NDC-AgNPs) were effective against CRAB by inhibiting the drug efflux mediated by the AdeB component of the AdeABC efflux pump. Similarly, the use of 3-O-substituted quercetin was shown to be effective both as an efflux pump inhibitor and as an inhibitor of metallo-beta-lactamases from carbapenem-resistant Enterobacteriaceae [142]. Natural compounds can also be used as effect enhancers for drugs with already-known antimicrobial activity. The use of resveratrol in combination with chlorhexidine exhibited strong synergistic activity against CRAB. However, the effect of resveratrol on efflux pumps is related to the inhibition of *adeB* gene expression, rather than the inhibition of pump activity, per se [143]. Furthermore, the combination of innovative compounds with already-known substances has generated encouraging results. For example, the regulation of genes associated with efflux pumps using cinnamon oil and trimethoprim managed to abolish carbapenem resistance in clinical isolates, pointing to a viable and practical solution to circumvent resistance [144].

Similarly to natural compounds, the chemical modification of already-known molecules also represents a promising strategy in the generation of new inhibitors. Inhibitors of *E. coli* AcrAB-TolC efflux pumps, namely 4-dihydroimidazoylanilinine and 4,6-diaminoquoniline, were chemically modified to act on the AdeIJK efflux pump of *A. baumannii*, showing good results and specificity [139]. Pyridylpiperazine, another recently discovered class of efflux pump inhibitors for *E. coli* [145], was also optimised by chemical modification to inhibit the activity of *A. baumannii* efflux pumps, particularly on AdeJ [146].

Another notable approach is the use of small molecules to enhance the efficacy of antimicrobials. *A. baumannii* has intrinsic resistance to fosfomycin due to the low uptake of this antibiotic and an active efflux mechanism mediated by the AbaF pump [147]. The molecule IITR08367 (bis(4-methylbenzyl) disulfide) was shown to be capable of increasing the antibacterial action of fosfomycin by acting as an inhibitor of the AbaF pump [148]. The discovery of this molecule may expand the potential of the therapeutic combinations available for the treatment of CRAB.

#### 3.4.2. Phage Therapy

Bacteriophage (or simply “phage”) therapy represents a promising alternative for the treatment of MDR infections, including those caused by CRAB, particularly in cases where conventional antibiotics have failed.

Despite its potential, research in this area remains limited, often confined to case reports (frequently involving patients with no alternative treatment options) [149,150]; small-scale studies conducted in vitro [151,152,153] or animal models [154]. One of the major challenges is the lack of standardisation across studies—different phages or phage cocktails are used, with varying dosages and treatment durations [155,156]. Although many reports show clinical improvement, this methodological heterogeneity impairs result comparability and hinders the development of standardised clinical protocols. Moreover, the possibility of a publication bias must be acknowledged, as studies with successful outcomes are more likely to be published, potentially altering the true efficacy of phage therapy. This issue must be addressed when considering the broader clinical applicability of this approach.

An additional concern is the limited understanding of bacterial resistance to phages. Without a thorough investigation into resistance mechanisms and evolutionary dynamics, there is a risk that phage therapy could mirror the trajectory of antibiotics, where misuse and overuse lead to the rapid loss of its effectiveness [157,158,159].

#### 3.4.3. Antimicrobial Peptides (AMPs)

Antimicrobial peptides (AMPs) have emerged as promising candidates in the fight against MDR pathogens, including *A. baumannii*. These peptides constitute a diverse class of short, cationic molecules produced by a wide range of organisms (e.g., bacteria, archaea, fungi, plants, and mammals) as a part of their innate immune defence. In addition to their antimicrobial properties, AMPs often exhibit immunomodulatory functions and have been associated with processes such as wound healing and apoptosis induction [160,161].

Multiple AMPs have demonstrated activity against *A. baumannii*, acting through distinct mechanisms. For example, LL-37 has been shown to disrupt bacterial membranes [162]; Mastoparan functions as a membrane-penetrating peptide [163]; ZY4 exerts its antimicrobial effects through membrane damage, pore formation, and increased permeability [164]; and P92 is a synthetic peptide targeting outer membrane protein A (AbOmpA) [165]. These varied mechanisms highlight the versatility of AMPs in targeting bacterial structures and functions. But despite encouraging in vitro results, many naturally occurring AMPs have failed to demonstrate consistent efficacy or safety in clinical trials, limiting their therapeutic application [166].

In light of the limitations associated with the clinical application of natural AMPs, recent research has increasingly focused on the rational design and engineering of novel peptides with enhanced pharmacological profiles. Synthetic, modified, or specifically targeted AMPs have demonstrated improved antimicrobial potency, greater structural stability, and increased selectivity toward bacterial cells [167]. These advancements represent a significant step forward in overcoming the shortcomings of naturally occurring peptides and may facilitate the successful translation of AMP-based therapies into clinical practice, particularly for the treatment of CRAB and other multidrug-resistant infections.

In this emerging field, where a growing number of AMP candidates are being investigated, organised information plays a crucial role in streamlining research efforts. One example is the recently published database, AbAMPdb, a curated resource compiling both natural and synthetic AMPs that are active against *A. baumannii,* which supports the identification and prioritisation of promising molecules for further preclinical evaluation [168].

#### 3.4.4. Gene Editing Tools

Gene editing technologies have emerged as promising strategies to combat antimicrobial resistance (AMR), particularly in CRAB. Among the principal tools are zinc finger nucleases (ZFNs), transcription activator-like effector nucleases (TALENs), and the CRISPR-Cas system. Of these, CRISPR-Cas has demonstrated the greatest potential due to its high precision, programmability, and ability to selectively target resistance genes. The current literature on CRISPR-Cas in AMR contexts primarily focuses on sensitising resistant bacteria to antibiotics by disrupting specific resistance determinants [169].

Several studies have demonstrated the applicability of exogenous or endogenous CRISPR-Cas systems in *A. baumannii*, allowing for the targeted disruption of AMR genes and or gene knockdown tools for the study of these genes’ mechanisms of action. One example is a study that utilises the clinical isolate AB43 of *A. baumannii*, which harbours a complete Type I-Fb CRISPR-Cas system. Specifically, CRISPR-Cas activity led to the downregulation of the quorum-sensing synthase abaI, resulting in reduced efflux pump expression, weakened biofilm formation, the increased production of reactive oxygen species, and ultimately, enhanced susceptibility to antibiotics. These findings indicate that targeting endogenous abaI through CRISPR-Cas may represent a novel strategy to potentiate bacterial sensitivity to antimicrobial agents [170].

In another study, a CRISPR interference (CRISPRi) system was developed to enable the transcriptional silencing of target genes in *A. baumannii*. The system proved effective in silencing target genes at the transcriptional level. Through its application, the role of the novel cell division protein AdvA was validated, and a previously uncharacterised AraC-family transcriptional regulator (ACX60_RS03245) was identified as critical for bacterial growth [171]. Additionally, a recent study reported the development of the sgRNA-I/L@ZS nanosystem (a nanobomb that delivered imipenem and CRISPR/Cas9), which was reported as a high-efficiency bactericide against CRAB [172].

Despite these advances, one of the major challenges in using CRISPR-Cas as a therapeutic tool is the efficient delivery of its components into bacterial cells. Two general strategies exist: CRISPR/Cas without delivery vehicles, e.g., leveraging endogenous systems; and CRISPR/Cas with delivery platforms, typically using bacteriophages or nanoparticles. Phage-based delivery, while promising, faces limitations such as a narrow host range, immunogenicity, and the lack of regulatory clarity. Modifying phages to expand their host specificity is a potential strategy to address these issues [173,174,175].

To overcome delivery limitations, nanotechnology has been integrated with CRISPR systems. In recent studies, liposomes and polymers have been used to encapsulate CRISPR-Cas components, protecting them from degradation and improving bacterial uptake, which led to increased knockout efficiency [173,174].

#### 3.4.5. Nanoparticle-Based Strategies

Nanoparticles (NPs) are nanometer-scale materials (sizes ranging from 1 to 100 nm) with unique physicochemical properties that enhance their interactions with bacterial cells. In antimicrobial resistance (AMR), NPs have been explored for their ability to bypass conventional resistance mechanisms through membrane disruption, reactive oxygen species (ROS) generation, and targeted drug delivery. Among the most studied NPs that can act as inherent therapies—e.g., titanium dioxide (TiO2) NPs, zinc oxide (Zn O) NPs [26], copper (Cu) NPs, nickel (Ni) NPs, selenium (Se) NPs, and silver (Ag) NPs—or as nano-carriers for antimicrobial agents “Nanobiotics” (e.g., liposomes) [176,177].

In a recent study, CuO NPs showed growth inhibition against MDR pathogens, including *A. baumannii*, with minimum inhibitory concentrations (MICs) ranging from 62.5 to 125 μg/mL. These nanoparticles also exhibited an antibiofilm potential and demonstrated antioxidant activity, enhancing their efficacy when combined with conventional antibiotics [178].

Although silver nanoparticles (nanosilver; NAg) are recognised as one of the most effective alternative antimicrobial agents, recent findings raise concerns about the potential for resistance development in *A. baumannii*. Prolonged exposure to NAg has been shown to induce stable physiological adaptations in *A. baumannii*, including alterations in biofilm formation and oxidative stress responses. These adaptations were associated with mutations in genes related to surface attachment and capsular polysaccharide biosynthesis. Moreover, a distinct tolerance phenotype was observed following exposure to the ionic form of silver (Ag^+^), marked by increased respiratory activity and morphological changes. These results underscore the capacity of *A. baumannii* to adapt to both nanoparticulate and ionic silver, highlighting the importance of the cautious and controlled use of NAg to preserve its efficacy and mitigate the risk of resistance emergence in clinical settings [179].

## 4. Computational Approaches Supporting the Fight Against Carbapenem-Resistant *A. baumannii*

### 4.1. AI-Based Screening and Design of Antimicrobials

Of all the applications described, the use of artificial intelligence (AI) is the one with the greatest disruptive potential. While AI is not a final product in itself, it serves as an enabling platform spanning diagnostic innovation, therapeutic design, and outbreak prevention. Decades before high-performance computing and AI became a reality, scientists were already suggesting that the 3D structure of proteins could be used in drug design [180]. The use of AI tools for protein structure determination, such as AlphaFold [181], has revolutionised structural biology, enabling the accurate prediction of protein structures within minutes with a high degree of reliability.

From a determined structure, it is possible to use other AI tools that analyse the surface of enzymes, such as beta-lactamases or transporters such as efflux pumps, and design molecules with an inhibitory potential [182,183]. For instance, in an in silico screening, five synthetic compounds were tested against three *A. baumannii* efflux pumps (AdeB, AdeG, and AdeJ). The compound KSA5, a novel imidazo[4,5-g]quinoline-4,9-dione derivative, stood out for its ability to inhibit antibiotic efflux. Molecular docking simulations revealed that KSA5 can bind to these efflux pumps by stably interacting with residues at the ligand binding site [184]). In another in silico screening study, 7500 molecules were tested for the growth inhibition of *A. baumannii* in vitro. These data were used to train a neural network model capable of predicting molecules with novel chemical structures and antibacterial activity against this pathogen. This computational strategy identified abaucin, a compound with selective activity against *A. baumannii*. Abaucin was shown to inhibit lipoprotein transport through interaction with the LolE protein. The therapeutic relevance was confirmed in an animal model, where abaucin demonstrated efficacy in the treatment of *A. baumannii* skin infections in mice [185]. A virtual screening of 11,648 natural compounds was performed to identify potential OmpW-targeting molecules in *A. baumannii*. Among these, demethoxycurcumin emerged as a top candidate due to its favourable pharmacokinetic properties and strong binding stability. Experimental validation demonstrated its antibacterial activity against multiple *A. baumannii* strains, including OmpW-deficient mutants, and revealed synergistic effects when combined with colistin [186].

Drug design has also been applied in the search for beta-lactamase inhibitors. A study seeking inhibitors for NDM-1 beta-lactamase designed 20 compounds based on naphthalene, thiazole, and sulfone molecules, which are capable of interacting with zinc ions at the enzyme’s active site. Using molecular docking, three promising compounds were identified, with an emphasis on T016, which showed high stability in molecular dynamics simulations and favourable pharmacokinetic properties, being a potential candidate for in vitro testing [187]. For AmpC inhibition, a study used deep machine learning to predict non-covalent inhibitors, employing classification models such as SVM, Random Forest, and neural networks. The models achieved up to 83% accuracy in identifying new inhibitors, allowing the analysis of the physicochemical characteristics and binding modes of these molecules [188].

### 4.2. AI for Genomic Interpretation and Phenotype Prediction

The evolution and reduction in the cost of DNA sequencing technologies have allowed the sequencing of thousands of bacterial genomes, with different resistance phenotypes, from various locations around the world. This large amount of genomic data has allowed the application of methodologies previously restricted to the study of human genomes, such as genome-wide association studies (GWASs) for bacteria [189]. GWASs are expanding the scope of resistance genotypes in *A. baumannii* beyond the classic resistance genes to include previously unrelated genes, such as glycosyltransferases and conserved hypothetical proteins [190].

Single-nucleotide polymorphisms (SNPs) at different positions in the genome can be used as indicators of resistance to a given antibiotic and used to train a resistance prediction network based on genomic data [191]. An AI tool trained to identify genomic patterns associated with resistance in conjunction with a rapid DNA sequencing methodology from blood samples (clinical metagenome) could revolutionise the point-of-care treatment of critical conditions, such as sepsis [192,193].

**Figure 3:**
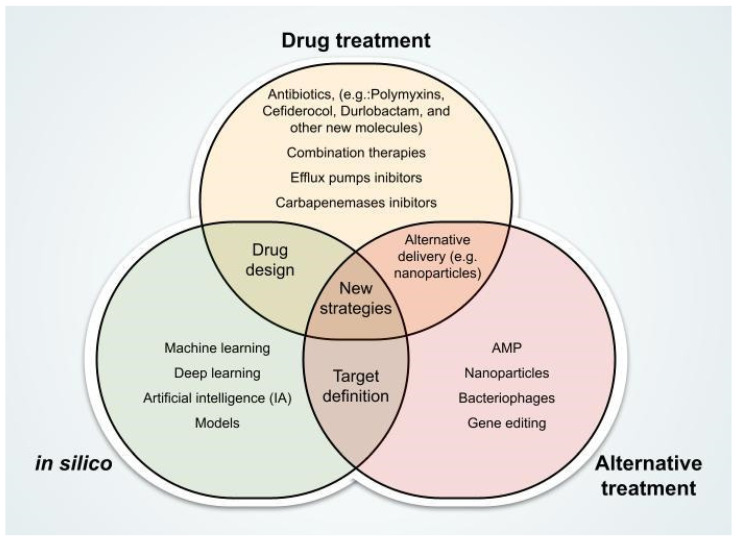
Integration of therapeutic and investigative strategies for CRAB through innovative and computational approaches. Abbreviation: AMP, antimicrobial peptides.

Furthermore, multi-omics studies that integrate genomic, transcriptomic, proteomic, lipidomic, and metabolomic data offer powerful opportunities to investigate in depth how bacteria respond to antibiotic exposure. The comprehensive integration of these datasets enhances our understanding of the adaptive plasticity of *A. baumannii*, facilitating not only the identification of novel pharmacological targets but also the development of more effective combination therapies [194]. However, the integration and interpretation of large-scale multi-omics data remain significant challenges. The application of AI, including machine learning algorithms, neural networks, and deep learning approaches, will undoubtedly be essential for advancing the field and unlocking the full potential of multi-omics analyses [195]. The multi-omics data can be used to create genome-scale metabolic models under different physiological conditions. The application of AI tools trained on metabolic models may provide suggestions for targets in central metabolic pathways that can be inactivated with bactericidal or bacteriostatic effects [196]. Figure 3 presents a Venn diagram depicting innovative strategies for CRAB treatment and investigation, along with their areas of overlap.

## 5. Conclusions

The growing prevalence of CRAB represents a threat to global public health, particularly in critical care settings, where therapeutic options are increasingly scarce. This review highlights the complex interplay of molecular mechanisms underlying resistance, including beta-lactamase production, outer membrane impermeability, efflux pump overexpression, and altered antibiotic targets, as well as the alarming global dissemination of resistance genes driven by mobile genetic elements. In this context, molecular surveillance, preferably through whole-genome sequencing, is critical. Large-scale genomic analyses have unveiled the dominance of highly resistant epidemic lineages, particularly the international clones IC1 and IC2, now reclassified as part of an epidemic super-lineage (ESL), which accounts for the majority of clinical CRAB isolates worldwide [21]. These clones exhibit the progressive acquisition of resistance genes and virulence traits, often driven by homologous recombination and transposable elements [22,23]. Importantly, genome-based approaches allow for the phylogenetic mapping of circulating strains and the temporal tracking of resistance mechanisms—tools essential for anticipating outbreaks, guiding empirical therapy, and informing public health interventions.

Despite these challenges, recent advances in antibiotic development, beta-lactamase inhibitors, and synergistic drug combinations offer renewed hope for treatment. Moreover, alternative approaches, such as bacteriophage therapy, antimicrobial peptides, nanoparticle-based delivery systems, and CRISPR-based gene editing, demonstrate significant promise, although further clinical validation is necessary. The integration of artificial intelligence and multi-omics technologies has opened new frontiers in understanding CRAB pathophysiology, enabling the identification of novel therapeutic targets and resistance biomarkers. Moving forward, the development of precision therapeutics and the implementation of robust surveillance systems—grounded in genomic and epidemiological data—will be essential to curbing the impact of CRAB and preserving the efficacy of last-line antimicrobials.

## Data Availability

No new data were created or analyzed in this study. Data sharing is not applicable to this article.
